# Leveraging Senescent Cancer Cell Membrane to Potentiate Cancer Immunotherapy Through Biomimetic Nanovaccine

**DOI:** 10.1002/advs.202400630

**Published:** 2024-06-12

**Authors:** Chao Yang, Yinglu Chen, Jie Liu, Wensheng Zhang, Yan He, Fangman Chen, Xiaochun Xie, Jie Tang, Shan Guan, Dan Shao, Zheng Wang, Liang Wang

**Affiliations:** ^1^ Department of Orthopedics Guangdong Provincial Key Laboratory of Bone and Joint Degeneration Diseases The Third Affiliated Hospital of Southern Medical University Guangzhou 510630 China; ^2^ National Engineering Research Center for Tissue Restoration and Reconstruction South China University of Technology Guangzhou Guangdong 510006 China; ^3^ School of Medicine South China University of Technology Guangzhou Guangdong 510006 China; ^4^ Drug Delivery, Disposition and Dynamics Monash Institute of Pharmaceutical Sciences Monash University Parkville VIC 3052 Australia; ^5^ National Engineering Research Center of Immunological Products Third Military Medical University Chongqing 400038 China; ^6^ CAS Key Laboratory of Nano‐Bio Interface Suzhou Institute of Nano‐Tech and NanoBionics Chinese Academy of Sciences Suzhou 215123 China

**Keywords:** biomimetic, engineered membrane, immunotherapy, nanovaccine, senescent cancer cell

## Abstract

Senescent cancer cells are endowed with high immunogenic potential that has been leveraged to elicit antitumor immunity and potentially complement anticancer therapies. However, the efficacy of live senescent cancer cell‐based vaccination is limited by interference from immunosuppressive senescence‐associated secretory phenotype and pro‐tumorigenic capacity of senescent cells. Here, a senescent cancer cell‐based nanovaccine with strong immunogenicity and favorable potential for immunotherapy is reported. The biomimetic nanovaccine integrating a senescent cancer cell membrane‐coated nanoadjuvant outperforms living senescent cancer cells in enhancing dendritic cells (DCs) internalization, improving lymph node targeting, and enhancing immune responses. In contrast to nanovaccines generated from immunogenic cell death‐induced tumor cells, senescent nanovaccines facilitate DC maturation, eliciting superior antitumor protection and improving therapeutic outcomes in melanoma‐challenged mice with fewer side effects when combined with αPD‐1. The study suggests a versatile biomanufacturing approach to maximize immunogenic potential and minimize adverse effects of senescent cancer cell‐based vaccination and advances the design of biomimetic nanovaccines for cancer immunotherapy.

## Graphical abstract

To overcome the limitations of senescent cancer cell‐based vaccines, a biomimetic strategy in which senescent cancer cell membranes are integrated with nanoadjuvants is created to increase the efficacy and safety of nanovaccines. In contrast to nanovaccines generated from immunogenic cell death‐induced cancer cells, senescent nanovaccines facilitate dendritic cell maturation, eliciting superior antitumor protection and improving therapeutic outcomes in melanoma‐challenged mice with fewer side effects when combined with αPD‐1.

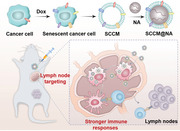

## 1 Introduction

Orchestrating immune system has demonstrated their clinical successes on battling a variety of previously untreatable malignancies through leveraging adaptive immune cell transfer, immune checkpoint inhibitors, and vaccines.^[^ ^1^ ^]^ Cancer vaccines, composed of tumor‐associated antigens (TAAs) and immunostimulatory molecules, harness immune cells to kill tumor cells with antigenicity adjuvanticity.^[^ ^2^ ^]^ Sophisticated nanovaccines are designed for targeted delivery of antigens and adjuvants, as well as modulation of the immune microenvironment.^[^ ^3^ ^]^ Instead of endowing such abilities through synthetic methods, researchers opt to use naturally derived cell membranes directly to create nanoplatforms with desirable biocompatibility, especially those that capture intrinsic functionalities inherited from donor cells.^[^ ^4^ ^]^ In this context, cancer cell membrane‐derived nanoparticles can provide a whole array of TAAs sourced either from autologous tumor tissues or established heterologous tumor cell lines, facilitating efficient antigen‐presenting cell (APC) targeting and personalized vaccination.^[^ ^5^ ^]^ Nevertheless, such biomimetic nanovaccines derived from pristine cancer cells contain inefficient membrane proteins and costimulatory components that can facilitate antigen recognition and presentation by APCs.^[^ ^6^ ^]^ Inspired by the fact that immunogenic cell death (ICD) in tumor cells can effectively induce a strong antitumor immune response, functionalized nanovaccines generated from ICD‐induced cancer cell membranes with high levels of calcinetin expression were found to trigger the uptake of APCs and boost immune activation.^[^ ^7^ ^]^ With these findings in mind, engineering biomimetic nanovaccines with desired active components can maximize their ability to promote effective antigen presentation and corresponding antitumoral immune response.

Senescence is a unique cellular response to cytotoxic regimens during cancer therapy.^[^ ^8^ ^]^ The production of the senescence‐associated secretory phenotype (SASP) allows senescent cancer cells to communicate stress signals with neighboring immune cells.^[^ ^9^ ^]^ In such a scenario, senescent cancer cells emerge as highly immunogenic entities, attributing their efficacy to the synergy of efficient antigens transfer, immunogenic SASPs release, and APCs activation, leading to efficient antitumor immune responses.^[^ ^10^ ^]^ With senescent cells demonstrating the ability to deliver antigens and stimulate APCs, senescent cancer cells outperform their pristine counterparts through enlisting innate and adaptive immune cells to support tumor regression.^[^ ^11^ ^]^ However, the long‐term vaccination of senescent cancer cells may cause protumorigenic outcomes. Increasing evidence suggests that the dynamic nature of SASPs and their ability to regulate immune responses in different ways are associated with positive or negative impacts on therapeutic outcomes.^[^ ^12^ ^]^ Conversely, senescent cancer cells also generate immunosuppressive SASPs that skew immature myeloid cells toward suppressive phenotypes, which may interfere with antigen presentation and activation of APCs during vaccination.^[^ ^13^ ^]^ Thus, leveraging effective antigen presentation with minimal interference from immunosuppressive SASPs and the possibility of rechallenge remain significant challenges in living senescent cancer cell‐based vaccination.

**Figure 1:**
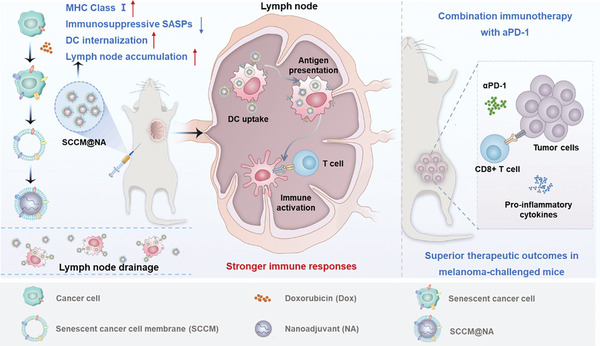
Schematic illustration of the biomimetic nanovaccines for enhanced DC internalization, improved lymph nodes targeting, and stronger immune responses.

To overcome the limitations of current senescent cancer cell‐based vaccines, we propose a biomimetic strategy in which senescent cancer cell membranes are integrated with nanoadjuvants to increase the efficacy and safety of nanovaccines (**Figure** **1**). In our study, senescent melanoma B16‐OVA cells were induced by long‐term incubation with a low dose of doxorubicin (DOX), and the engineered whole membranes were coated onto cytosinephosphate‐guanosine oligodeoxynucleotide (CpG)‐loaded biodegradable nanoadjuvant to yield a biomimetic nanovaccine (SCCM@NA). We demonstrated the advantages of utilizing a senescent cancer cell membrane in a context in which it could enable enhanced antigen presentation, improved lymph node accumulation, and stronger immune responses for biomimetic nanovaccines design when compared with living senescent cancer cell‐based vaccines. We then compared the immunogenic potential of these nanovaccines derived from cancer cells under senescence or ICD and observed that senescent cancer cell membrane‐based nanovaccines were more efficient at activating APCs and eliciting antitumor protection in a prophylactic setting. Furthermore, when combined with αPD‐1 checkpoint blockade, personalized senescent cancer cell membrane‐based nanovaccines exhibited a profound enhancement in the therapeutic outcomes for mice challenged with melanoma. Our study offers a biomimetic immunoengineering strategy to facilitate cancer immunotherapy.

## 2 Results and Discussion

**Figure 2:**
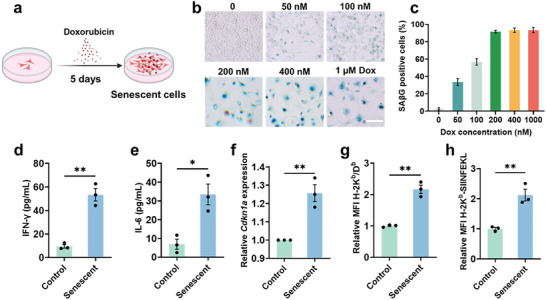
Induction and identification of senescent cells. a) Scheme of inducing cellular senescence. b) SAβG staining of B16‐OVA cancer cells following senescence induction via treatment with a series of concentrations of Dox over a 5‐day period. Scale bar, 100 µm. c) Percentage of the SAβG‐positive cells in (b). Expression levels of IFN‐γ d) and IL‐6 e) in the media of normal or senescent cells. f) The mRNA expression level of *Cdkn1a* in untreated and senescent B16‐OVA cancer cells. The H‐2K^b^/D^b^ expression g) and the presentation of the OVA‐derived peptide SIINFEKL bound to H‐2K^b^ h) were assessed in untreated and senescent B16‐OVA cancer cells. All data are mean ± SD; n = 3. ^*^ *P* < 0.05, ^**^ *P* < 0.01.

To induce senescence in B16‐OVA melanoma cells, cells were subjected to incubation with varying concentrations of DOX over a 5‐day period (**Figure** **2**a). Evaluation through senescence‐associated beta‐galactosidase (SAβG) staining revealed that 200 nM DOX effectively promoted the onset of cellular senescence with decreased toxicity (Figure 2b,c; Figure S1, Supporting Information). In this case, markedly increased protein levels were associated with SASP markers, including interferon‐gamma (IFN‐γ), interleukin‐6 (IL‐6) and tumor necrosis factor‐alpha (TNF‐α) (Figure 2d,e; Figure S1, Supporting Information), and with enhanced mRNA expression of the senescence‐associated gene *Cdkn1a* (Figure 2f), suggesting the success of inducing senescent cancer cells. Given that elevated major histocompatibility complex class I (MHC‐I) antigen processing and presentation are classic characteristics of senescent cells,^[^ ^11^ ^]^ our investigation delved into the expression levels of MHC‐I molecules on senescent B16‐OVA cells via determination of H‐2K^b^/D^b^ markers and the H‐2K^b^‐restricted ovalbumin (OVA)‐derived peptide SIINFEKL. As shown in Figure 2g,h, senescent cancer cells presented markedly greater amounts of both MHC‐I and OVA‐specific SIINFEKL bound to H‐2K^b^ than did their normal counterparts. Taken together, these findings demonstrated that DOX‐induced melanoma B16‐OVA cells developed classic senescent characteristics.

**Figure 3:**
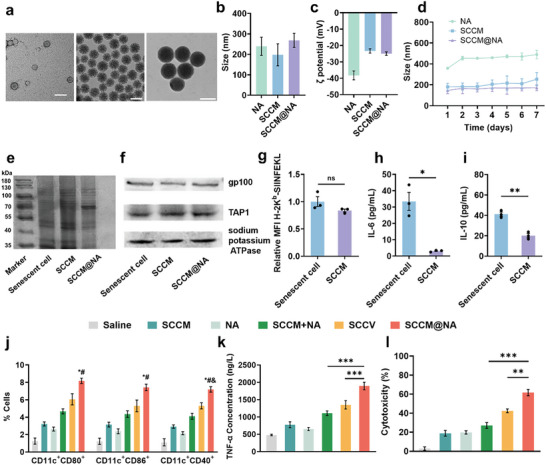
Characterization of biomimetic nanovaccines. a) The TEM images of SCCM vesicles (left), NA (middle), and SCCM@NA (right). Scale bar, 200 nm. Hydrodynamic sizes b) and ζ potential c) of NA, SCCM vesicles, and SCCM@NA. d) Long‐term colloidal stability of the NA, SCCM vesicles, and SCCM@NA nanoparticles stored in PBS for 7 days at 4 °C. e) SDS‐PAGE protein analysis of senescent cell lysates, SCCM, and SCCM@NA. f) gp100 and TAP1 expression in senescent cells, SCCM and SCCM@NA. g) The OVA‐derived peptide SIINFEKL bound to H‐2K^b^ presentation in senescent B16‐OVA cells and SCCM vesicles. Expression levels of IL‐6 h) and IL‐10 i) in senescent B16‐OVA cancer cells and SCCM vesicles. j) Effect of various treatments on DC maturation in vitro. Quantitative analysis for proportion of CD11c+CD80+, CD11c+CD86+, and CD11c+CD40+ cells. ^*^ *P* < 0.01 versus SCCM group, ^#^ *P* < 0.01 versus NA group, &*P* < 0.05 versus SCCV group. Secretion of TNF‐α k) in BMDC suspensions was quantified using ELISA. l) The cytotoxicity of T cells against B16‐OVA cells after co‐incubation with BMDCs activated by different nanovaccines. All data are mean ± SD; n = 3. ^*^ *P* < 0.05, ^**^ *P* < 0.01, ^***^ *P* < 0.001, and *ns*, not significant.

Next, we collected whole membranes from senescent B16‐OVA cells and coated them with biodegradable nanoadjuvants (NA), which consisted of CpG‐encapsulated diselenide‐bridged mesoporous silica nanoparticles that were degradable in a redox‐responsive manner and subsequently released (Figure S2, Supporting Information). The CpG‐loading content was calculated to be 9.7 µg of CpG per 1 mg of diselenide‐bridged mesoporous silica nanoparticles. The Brunauer–Emmett–Teller (BET) surface area, pore diameter and pore volume of NA were found to be 612.5 m^2^ g^−1^, 7.9 nm, and 1.2 cm^3^ g^−1^, respectively (Figure S3, Supporting Information). As shown in the electron microscopy images (**Figure** **3**a; Figure S2, Supporting Information), the biomimetic nanovaccines (SCCM@NA) exhibited a consistently spherical morphology characterized by a distinctive core‐shell structure, setting them apart from the bare NA cores. Dynamic light scattering analysis revealed that SCCM@NA particles exhibited a larger hydrodynamic size compared to NA particles (Figure 3b). The zeta potential of SCCM@NA was observed to be −24.9 mV (Figure 3c), which was closer to that of negatively charged cell membrane vesicles (−23.3 mV). The hydrodynamic diameter of SCCM@NA maintained in phosphate‐buffered saline (PBS) was monitored for 7 days (Figure 3d), indicating that the biomimetic nanovaccines possessed long‐term colloidal stability. Protein electrophoresis demonstrated effective retention of membrane proteins from the source senescent cell membranes on SCCM@NA after coating (Figure 3e). Western blot analysis further confirmed the well‐preserved and specific expression of both gp100 and TAP1, derived from the source senescent cancer cell membranes, on SCCM@NA (Figure 3f), collectively demonstrating the successful coating of senescent cancer cell membranes. Next, we investigated whether MHC‐I and SASPs were preserved after coating senescent cancer cell membranes. As expected, more than 80% of the OVA‐derived peptide SIINFEKL were present on SCCM vesicles (Figure 3g; Figure S4, Supporting Information), indicating the vesicles derived from senescent cancer cells remained the high immunogenicity. In contrast, neither proinflammatory SASP‐related IL‐6 nor anti‐inflammatory SASP‐related IL‐10 were found in SCCM vesicles (Figure 3h,i), suggesting that the SCCM vesicles exhibited no interference from immunosuppressive SASPs from senescent cancer cells.

Given that the biocompatibility of nanovaccines is critical for their clinical application,^[^ ^6b^ ^]^ low cytotoxicity was found when the concentration of nanovaccines was less than 100 µg mL^−1^ in two typical types of APCs (BMDCs and RAW 264.7 cells) (Figure S5, Supporting Information). We then compared the dendritic cell (DC) maturation effects of SCCM@NA and senescent cancer cell‐based vaccine (SCCV) through assessing the expression levels of costimulatory markers CD80, CD86, and CD40, as well as the secretion of TNF‐α and IL‐6. Interestingly, compared with SCCV, SCCM@NA markedly increased the expression of CD80, CD86, and CD40 (Figure 3j). Consistently, among all the tested vaccines, SCCM@NA‐immunized macrophages and DCs exhibited the highest levels of TNF‐α and IL‐6 secretion, respectively (Figure 3k; Figure S6, Supporting Information), along with the greatest extent of tumor cell killing by CD8+ T cells (Figure 3l). Notably, SCCM@NA led to more DC activation than did the bulk mixture of SCCM and NA or their single compartment, which might be attributed to efficient codelivery of adjuvants and antigens.

**Figure 4:**
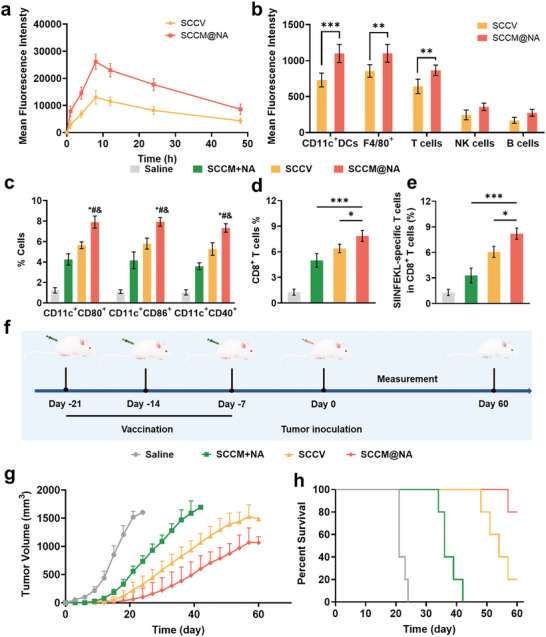
Lymph nodes targeting and prophylactic effects of the biomimetic nanovaccines. a) Quantitative analysis of fluorescence signals emanating from DiR‐labeled nanovaccines within lymph nodes (n = 3). b) Uptake profile of DiR‐labeled nanovaccines by key immune cell populations, including DCs, macrophages, T cells, NK cells, and B cells within lymph nodes at 24 h post‐injection (n = 3). c) Effect of various treatments on DC maturation. Quantitative analysis for proportion of CD11c+CD80+, CD11c+CD86+, and CD11c+CD40+ cells within the popliteal lymph nodes (n = 3). ^*^ *P* < 0.05 versus Saline group, ^#^ *P* < 0.05 versus SCCM+NA group, &*P* < 0.05 versus SCCV group. d) Measurement of the induction of CD8+ T cells generation within spleens post‐vaccination (n = 3). e) Quantitative assay for CD8+ T cells specific to the SIINFEKL antigen (n = 3). f) Illustrative depiction of the prophylactic experimental design and methodology. g) Quantitative assay of tumors volume (n = 5). h) Survival profiles of mice after immunization with different formulations (n = 5). All data are mean ± SD. ^*^ *P* < 0.05, ^**^ *P* < 0.01, and ^***^ *P* < 0.001.

Inspired by effective DC maturation and T‐cell activation with biomimetic senescent nanovaccines as opposed to senescent cancer cell‐based vaccines in vitro, we examined the efficacy of subcutaneous injection of DiR‐labeled membrane‐derived vaccines in promoting lymphatic drainage. Compared to that in the SCCV group, in the SCCM@NA group, the accumulation peaked at 8 h post administration, and rapid accumulation was observed in lymph nodes at 1 h post injection (**Figure** **4**a; Figure S7, Supporting Information). In terms of targeting APCs internalization, both DCs and macrophages in the lymph nodes preferred endocytosing SCCM@NA to SCCV, indicating increased recognition of tumor antigens by the APCs (Figure 4b). Additionally, SCCM@NA was localized to APCs and T cells but not to NK cells or B cells in the lymph nodes. Like in vitro results, SCCM@NA outperformed the bulk mixture of SCCM and NA on the basis of DC activation in lymph nodes (Figure 4c). Consistently, mice immunized with SCCM@NA displayed higher counts of SIINFEKL‐specific CD8+ T cells compared to those immunized with SCCV (Figure 4d,e). Next, we investigated the prophylactic effects of these compounds in a B16‐OVA melanoma‐challenged murine model. C57BL/6 mice were subcutaneously injected with different types of nanovaccines on Days 21, 14, and 7, followed by inoculated with B16‐OVA cells on Day 0 (Figure 4f). As shown in Figure 4g,h, both SCCM@NA and SCCV significantly delayed tumor growth compared with SCCM plus NA, but SCCM@NA had the greatest inhibitory effect and longest survival time (over 60 days). Collectively, these findings revealed that, compared with senescent cancer cell‐based vaccines, biomimetic senescent nanovaccines had profound immunological antitumor effects.

**Figure 5:**
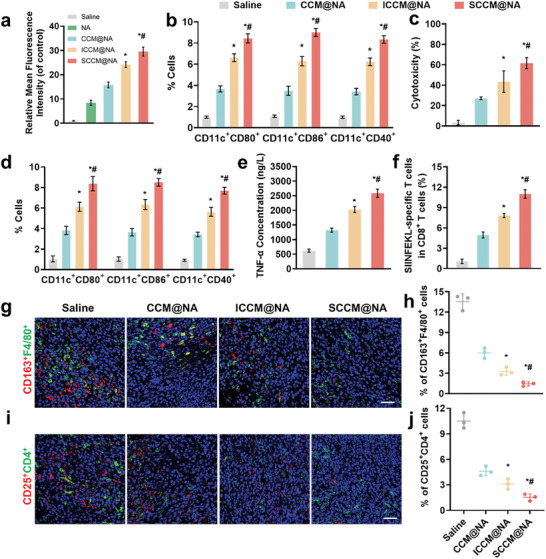
Comparison of the effects of three biomimetic nanovaccines on DC activation and immune responses. a) The cellular internalization of FITC‐labeled NA, CCM@NA, SCCM@NA, and ICCM@NA in BMDCs following a 1‐h exposure. b) Effect of various treatments on DC maturation in vitro. Quantitative analysis for percentage of CD11c+CD80+, CD11c+CD86+, and CD11c+CD40+ cells (n = 3). c) The cytotoxicity of T cells against B16‐OVA cells after co‐incubation with BMDCs activated by different nanovaccines. d) Effect of various treatments on DC maturation. Quantitative analysis for proportion of CD11c+CD80+, CD11c+CD86+, and CD11c+CD40+ cells within the popliteal lymph nodes (n = 3). e) Quantitative analysis of TNF‐α secretion. f) Quantitative analysis of CD8+ T cells specific to the SIINFEKL antigen. g) Immunohistochemistry analysis of M‐2 macrophages in tumor tissues, represented by CD163 (red) and F4/80 (green). i) Immunohistochemistry analysis of Tregs in tumor tissues, represented by CD25 (red) and CD4 (green). Scale bar, 100 µm. The calculated ratios of M‐2 macrophages h) and Tregs j) relative to the whole cells count within the corresponding tissue area. All data are mean ± SD; n = 3. ^*^ *P* < 0.05 versus CCM@NA group, #*P* < 0.05 versus ICCM@NA group.

Based on these findings, we investigated whether biomimetic nanovaccines derived from senescent cancer cells had immunogenic advantages toward their compartments from ICD‐induced cancer cells or untreated cancer cells. Having fabricated an ICD‐induced cancer cell membrane‐coated nanoadjuvant (ICCM@NA) and a cancer cell membrane‐coated nanoadjuvant (CCM@NA) (Figures S8 and S9, Supporting Information), we identified that all nanovaccines showed higher intracellular internalization with DCs compared with bare NA (**Figure** **5**a; Figure S10, Supporting Information), which was likely because of the higher stability and the easier internalization of phospholipids after cell membrane coating, suggesting that the cell membrane coating might improve the affinity of DCs to nanovaccines. Furthermore, both SCCM@NA and ICCM@NA presented greater DC uptake efficiency than CCM@NA did, which might be explained by the greater exposure of danger signals on the surface of those engineered cell membranes. Notably, DCs captured SCCM@NA more efficiently than ICCM@NA, suggesting that biomimetic senescent nanovaccines might better present antigens and adjuvants to APCs. Similarly, there were greater levels of DC maturation and T cell activation in vitro after incubation with SCCM@NA than after incubation with ICCM@NA or CCM@NA (Figure 5b,c; Figure S11, Supporting Information). In parallel with the in vitro findings, SCCM@NA‐treated cells produced the greatest proportion of mature DCs in lymph nodes; produced the highest levels of secreted TNF‐α, IFN‐β, IL‐6, and IL‐12; and generated more T cells specific for OVA (Figure 5d–f; Figure S12, Supporting Information). Although the DC‐T‐cell axis plays a vital role in eliciting an antitumor immune response,^[^ ^14^ ^]^ it is essential to understand the immunosuppressive TME for facilitating therapeutic outcomes. We thus investigated the tumor infiltration of immunosuppressive cells, including M‐2 macrophages and regulatory T cells (Tregs), in mice following immunization with different biomimetic formulations. The least count of M‐2 macrophages and Tregs was observed in the tumors of B16‐OVA tumor‐bearing mice subsequent to vaccination with SCCM@NA (Figure 5g–j), indicating that biomimetic senescent nanovaccines exhibited greater potential to remodel the immunosuppressive TME than other biomimetic nanovaccines.

**Figure 6:**
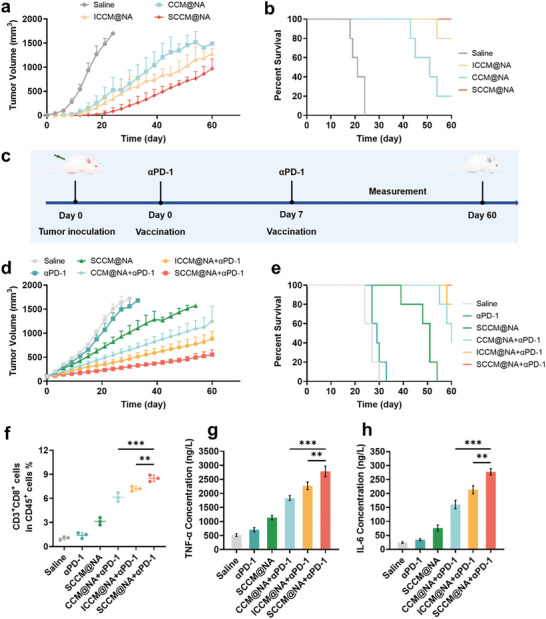
Prophylactic and therapeutic effects of biomimetic nanovaccines. a) Quantitative assay of tumors volume (n = 5). b) Survival profiles of B16‐OVA tumors bearing mice following immunization with different biomimetic nanovaccines (n = 5). c) Illustrative depiction of the therapeutic experimental design and methodology. d) Quantitative assay of tumors volume (n = 5). e) Survival profiles of B16‐OVA tumors bearing mice after immunization with different formulations (n = 5). f) Measurement of the induction of CD8+ T cells generation post‐immunization (n = 3). Quantitative assay of TNF‐α g) and IL‐6 h) secretion via ELISA (n = 3). All data are mean ± SD. ^**^ *P* < 0.01, and ^***^ *P* < 0.001.

Having demonstrated the immunogenic benefits of biomimetic senescent nanovaccines, we investigated prophylactic B16‐OVA‐treated murine melanoma models, aiming to evaluate protective antitumor immune responses. As shown in **Figure** **6**a,b, vaccination with three biomimetic nanovaccines significantly delayed tumor growth and prolonged survival in mice as compared to the control group, whereas the tumor progression effect and survival rate improvement were greater in the SCCM@NA group than in the ICCM@NA and CCM@NA groups. Encouraged by these promising findings, we next investigated whether biomimetic senescent nanovaccines could elicit potent therapeutic efficacy when combined with an immune checkpoint blockade strategy in established B16 murine melanoma models. Mice were administered B16‐OVA cells and subsequently immunized with nanovaccines on two occasions on Day 0 and Day 7 following the tumor reaching a volume of 100 mm^3^ (Figure 6c; Figure S13, Supporting Information). As shown in Figure 6d,e, αPD‐1 alone failed to inhibit tumor progression or improve the survival of mice. In contrast, SCCM@NA plus αPD‐1 notably suppressed tumor growth and extended the median survival of mice to more than 60 days, which was better than that of SCCM@NA alone and ICCM@NA plus αPD‐1. Consistently, the combination of SCCM@NA and αPD‐1 triggered the greatest increase in CD8+ T cells and in the secretion of most proinflammatory cytokines, including TNF‐α, IL‐6, and IFN‐β (Figure 6f–h; Figure S14, Supporting Information). Together, such combined immunotherapy has resulted in a remarkable therapeutic outcome for suppressing the development of melanoma. Additionally, there were no changes in terms of body weight, serum biochemical serum biochemistry indexes, or histopathology of the heart, liver, spleen, lung or kidney in any of the treatment groups compared to the control group (Figures S15–S17, Supporting Information), indicating the low systemic toxicity associated with biomimetic senescent nanovaccine‐based immunotherapy.

## 3 Conclusion

Although the immunogenic potential of senescent cancer cells has been leveraged to elicit antitumor immunity and potentially complement anticancer therapies, this strategy should be carefully orchestrated to avoid their immunosuppressive and pro‐tumorigenic capacities. In this study, we engineered a biomimetic nanovaccine derived from senescent cancer cell membrane to achieve an efficient antitumor response. Such a biomimetic senescent nanovaccine outperformed senescent cancer cell‐based vaccines in several ways, such as enhancing DC internalization, improving lymph nodes targeting and enhancing immune responses, and overcoming the possible interference from immunosuppressive SASPs on whole senescent cancer cells. Compared with biomimetic ICD‐mediated nanovaccines, biomimetic senescent nanovaccines exhibited enhanced APCs maturation and immune activation. Consequently, biomimetic senescent nanovaccines demonstrated not only optimal prophylactic efficacy but also served as a strong therapeutic platform, markedly inhibiting the growth of pre‐established melanoma tumors with decreased systemic toxicity when combined with αPD‐1. Overall, the current biomimetic nanovaccines engineered with senescent cancer cell membranes are potential candidates for improving immune response and cancer immunotherapy. Owing to their unique advantages in facilitating antigen presentation and APCs maturation, these cells might represent a promising tool for improving vaccination strategies for efficient and safe cancer immunotherapy. Thus, we suggest that biomimetic nanovaccines can be employed as promising personalized cancer vaccines through the development of senescent cell membranes from tumor tissues after surgery. Beyond cancer, a spectrum of inflammatory and aging‐associated diseases is driven, in part, by the presence of senescent cells. It is enticing to speculate that tailoring the design of biomimetic nanovaccines based on senescent cells might have therapeutic benefits in fruitful directions.

## 4 Experimental Section

### Senescence and ICD Induction

Senescence was induced through incubation with doxorubicin at a series of concentration for 5 days. Following this treatment period, senescent cells were harvested and used for subsequent experiments. Induction of ICD was achieved by subjecting cells to a high dose of doxorubicin (5 µM) for a period of 18 h.

### SAβG Assay

The induced senescent cells underwent a series of procedures: they were first washed with fresh PBS, followed by fixation for 15 min. Afterward, they were washed three times with PBS, and subjected to overnight incubation at 37 °C in a staining solution containing 1 mg/mL X‐Gal (Beyotime, C0602). The cells were then washed with PBS and examined for visualization using a Leica DM750 microscope.

### Synthesis of Biomimetic Nanovaccines

The synthesis of diselenide‐bridged mesoporous silica nanoparticles commenced by combining 0.15 g of triethanolamine (TEAH3) and 0.6 g of CTAB in 50 mL of deionized water at 80 °C, with mild stirring for 30 min. Subsequently, 1.5 g of bis(3‐(triethoxysilyl) propyl) diselenide (BTESePD) and 6 g of TEOS were added to the solution and reacted for an additional 5 h. The products were subsequently obtained via 8000 rpm centrifugation for 10 min and washed with deionized water for three times. To extract the CTAB solution and facilitate the formation of a mesoporous structure, the resulting products were supplemented with 80 mL of an ethanol solution of NH_4_NO_3_ (1.5% w/v) and refluxed at 105 °C for 8 h. Then, the diselenide‐bridged mesoporous silica nanoparticles were isolated by centrifugation and stored at 4 °C according to the previous reported methods.^[^ ^15^ ^]^

To preload CpG, 100 µg CpG was mixed with 1 mg diselenide‐bridged mesoporous silica nanoparticles. After 24 h of stirring, the CpG‐encapsulated diselenide‐bridged mesoporous silica nanoparticles (NA) were by centrifugation at 8000 rpm for 10 min and the unbound CpG was quantified by UV–vis.^[^ ^5^, ^16^ ^]^

To extract cell membrane vesicles, 1 × 10^7^ untreated B16‐OVA cells, ICD‐B16‐OVA cells, and senescent B16‐OVA cells were suspended in 10 mL of hypotonic lysis buffer and disrupted using a Dounce homogenizer, followed by a sequential centrifugation. The obtained ghosts were resuspended in 1 mL of deionized water with sonication for 5 min, and then extruded serially through successive polycarbonate membranes (Whatman) using an Avanti Mini‐Extruder (Avanti Polar Lipids) to form cell membrane vesicles. To prepare nanovaccines, NA were mixed with CM vesicles in deionized water, sonicated for 10 min, and then extruded serially through polycarbonate membranes. The products were collected after centrifugation and then stored at 4 °C.

### DC and Macrophage Activation

BMDCs were plated in a 6‐well plate at a density of 2 × 10^6^ cells in BMDC growth media overnight. Then, the BMDCs were treated with NA, SCCM, SCCV, CCM@NA, ICCM@NA, SCCM@NA, SCCM vesicle plus NA at 12.5 µg mL^−1^ or an equal dose of senescent cells and co‐incubated for 12 h. The cells were then washed with fresh media and cultured for additional 48 h. Subsequently, the cells were trypsinized, resuspended, and then stained with FITC‐conjugated anti‐mouse CD11c as well as APC‐conjugated anti‐mouse CD80, CD86 or CD40 for flow cytometry analysis. Data were obtained using a flow cytometer (FongCyte, Beijing Challen Biotechnology Co., Ltd) and analyzed using the FlowJo software.

To investigate the TNF‐α and IL‐6 secretion, BMDCs were incubated with NA, SCCM, SCCV, CCM@NA, ICCM@NA, SCCM@NA, or SCCM vesicle plus NA for 12 h. Then, the cells were washed with fresh medium and cultured for an addition of 48 h. Afterward, the culture medium was harvested, and the cytokine content was quantified using ELISA kits.

RAW 264.7 cells were plated in a 6‐well plate at a density of 5 × 10^5^ cells in complete growth media overnight. Then, RAW 264.7 cells were treated with NA, SCCM, SCCV, SCCM@NA, SCCM vesicle plus NA at 12.5 µg mL^−1^ or an equal dose of senescent cells and co‐incubated for 24 h. Then cell supernatants were harvested and cytokine content was quantified using TNF‐α ELISA kits.

To evaluate DC activation in vivo, C57BL/6J mice were received subcutaneous injections of saline, SCCV, CCM@NA, ICCM@NA, SCCM@NA, or SCCM vesicle plus NA into the footpad at a dosage of 5 mg kg^−1^ NA or an equivalent dose of senescent cells. After 24 h of injection, popliteal lymph was harvested nodes, dissociated by pipetting, and stained with FITC‐conjugated anti‐mouse CD11c, as well as APC‐conjugated anti‐mouse CD80, CD86 or CD40. Then, the expression levels of CD80, CD86 or CD40 on the DCs were measured using flow cytometry.

To measure the secretion of proinflammatory cytokines, lymph node‐derived cells were harvested at 24 h post‐injection with various formulations. Then, these single‐cells were cultured within BMDC growth media. After 48 h incubation, the cell supernatants were harvested and analyzed using TNF‐α, IL‐6, IL‐12, and IFN‐β ELISA kits.

### In Vitro Assessment of CD8+ T Cells Toxicity

To determine the toxicity of CD8+ T cells, they were obtained from the spleen of C57BL/6 mice using a CD8a microbeads isolation kit. These cells were then cultured in RPMI‐1640 medium supplemented with 10% FBS, 1% penicillin/streptomycin, 5 µg/mL anti‐CD3, 1 µg mL^−1^ anti‐CD28, and 10 ng mL^−1^ IL‐2. Subsequently, the CD8+ T cells were co‐cultured with pre‐treated BMDCs at a 1:1 cell ratio for 3 days. Following this incubation period, the stimulated CD8+ T cells were harvested and exposed to target B16‐OVA cells (5 × 10^3^ per well) in 96‐well plates, at a cell ratio of 10:1. After 48 h incubation, the level of lactic dehydrogenase (LDH) leakage, signifying the cytotoxicity against cancer cells of the activated CD8+ T cells, was assessed using an LDH detection kit as per the provided instructions.

### Animal Experiments

All animal experiments were conducted in agreement with the guidelines outlined in the Guide for the Care and Use of Laboratory Animals, and the procedures were approved by the Institutional Animal Care and Use Committee of South China University of Technology (Guangzhou, China) (approval No.2023061) and the Suzhou Institute of Biomedical Engineering and Technology Chinese Academy of Sciences Animal Care and Use Committee. C57BL/6J mice at 6 weeks old were purchased from Hunan SJA Laboratory Animal Co., Ltd.

### In Vivo T Cell Activation

C57BL/6J mice were received subcutaneous injections of saline, SCCV, CCM@NA, ICCM@NA, SCCM@NA, SCCM vesicle plus NA into the foot pad at 5 mg kg^−1^ NA or an equal dose of senescent cells. Collected spleens and processed them into cell suspensions. Following the eliminating of red blood cells through lysis, splenocytes were washed in PBS, resuspended, and then stained with PE‐labeled H‐2K^b^ OVA tetramer, as well as APC‐conjugated anti‐mouse CD8a antibody. These labeled splenocytes were then assessed for the proportions of SIINFEKL‐specific CD8+ T cells utilizing the tetramer staining method as outlined in the standard protocol.

### Lymph Node Accumulation

C57BL/6J mice received subcutaneous injections of DiR‐labeled SCCV or SCCM@NA into the footpad. Then, the mice were euthanized, and collected the popliteal lymph nodes at the predetermined times. Subsequently, the lymph nodes were detected by using a Bruker In Vivo Imaging System. To explore cellular internalization in the lymph nodes, the lymph nodes were dissociated 24 h after subcutaneous injection by pipetting. Then, the macrophages and DCs were stained with an F4/80 monoclonal antibody and a CD11c monoclonal antibody, respectively, for 30 min. The cells were subsequently analyzed via FACS.

### Prophylactic Effect

C57BL/6J mice received subcutaneous injections of saline, SCCV, SCCM vesicles plus NA, or SCCM@NA every 7 days for a total of three administrations. One week after the last administration, 2 × 10^4^ B16‐OVA cells were introduced by subcutaneous injection into the right flanks of the mice. The tumor size was monitored every two days, and the tumor volume was determined using the following formula: tumor volume (mm^3^) = π/6 ×length × width.^2^ The mice were euthanized upon reaching a tumor volume of 1500 mm^3^. The survival days of all the mice were recorded.

C57BL/6J mice received subcutaneous injections of saline, CCM@NA, ICCM@NA, or SCCM@NA every 7 days for a total of three administrations. 1 week after the last administration, 2 × 10^4^ B16‐OVA cells were introduced by subcutaneous injection into the right flanks of the mice. The tumor size was monitored every two days, and the tumor volume was determined using the following formula: tumor volume (mm^3^) = π/6 ×length × width.^2^ The mice were euthanized upon reaching a tumor volume of 1500 mm^3^. The survival days of all the mice were recorded.

### Therapeutic Effect

A total of 1 × 10^5^ B16‐OVA cells were introduced by subcutaneous injection into the right flanks of C57BL/6J mice. Upon reaching a xenograft volume of 100 mm^3^, the tumor‐bearing mice were randomly assigned to different six groups and administered saline, αPD‐1, SCCM@NA, CCM@NA plus αPD‐1, ICCM@NA plus αPD‐1 or SCCM@NA plus αPD‐1. In the nanovaccine treatment groups, mice received a subcutaneous injection of 100 µL of nanovaccines (5 mg kg^−1^) on Day 0 and 7 after the tumor volume reached 100 mm^3^. In the treatment αPD‐1 groups, the mice received a subcutaneous injection of αPD‐1 at the dose of 1 mg kg^−1^ on Day 0 and 7. The tumor sizes were recorded and calculated every two days. The mice were euthanized upon reaching a tumor volume of 1500 mm^3^. The survival days of the mice were recorded.

### Statistical Analysis

Statistical distinctions among groups were evaluated employing the unpaired Student's *t*‐test for comparisons involving two samples and one‐way analysis of variance (ANOVA) with Tukey's post hoc test for comparisons involving multiple samples. These statistical analyses were conducted using GraphPad Prism 8 software.

## Conflict of Interest

The authors declare no conflicts of interest.

## Author Contributions

C.Y. and Y.C. contributed equally to this work. C.Y., Y.C., J.L., W.Z., Y.H., F.C., and X.X. performed the experiments; C.Y., Y.C., J.T., and S.G. performed data interpretation, and drafted the manuscript; D.S., Z.W., and L.W. conceived and supervised the project and revised the manuscript.

## Supporting information

Supporting Information

## Data Availability

The data that support the findings of this study are available on request from the corresponding author. The data are not publicly available due to privacy or ethical restrictions.
