# Supplementary material for: Leveraging Senescent Cancer Cell Membrane to Potentiate Cancer Immunotherapy Through Biomimetic Nanovaccine
Source: Adv Sci (Weinh). 2024 Jun 12;11(30):2400630. doi: 10.1002/advs.202400630 (PMC11321648; doi:10.1002/advs.202400630)
Supplement: Supplementary file 1 — Supporting Information [file ADVS-11-2400630-s001.docx]

Supporting Information

Leveraging Senescent Cancer Cell Membrane to Potentiate Cancer Immunotherapy Through Biomimetic Nanovaccine

Chao Yang, Yinglu Chen, Jie Liu, Wensheng Zhang, Yan He, Fangman Chen, Xiaochun Xie, Jie Tang, Shan Guan, Dan Shao*, Zheng Wang*, and Liang Wang*

C. Yang, J. Liu, W. Zhang, Prof. L. Wang

Department of Orthopedics, Guangdong Provincial Key Laboratory of Bone and Joint Degeneration Diseases, The Third Affiliated Hospital of Southern Medical University, Guangzhou 510630, China.

E-mail: wl3388@smu.edu.cn

Y. Chen, Y. He, F. Chen, X. Xie, Prof. D. Shao

National Engineering Research Center for Tissue Restoration and Reconstruction, South China University of Technology, Guangzhou, Guangdong 510006, China

E-mail: shaodan@scut.edu.cn

Y. Chen, X. Xie, Prof. D. Shao

School of Medicine, South China University of Technology Guangzhou, Guangdong

Prof. Z. Wang

CAS Key Laboratory of Nano-Bio Interface, Suzhou Institute of Nano-Tech and NanoBionics, Chinese Academy of Sciences, Suzhou 215123, China

E-mail: zwang2021@sinano.ac.cn

Prof. J. Tang

Drug Delivery, Disposition and Dynamics, Monash Institute of Pharmaceutical Sciences, Monash University, Parkville, VIC 3052, Australia

Prof. S. Guan

National Engineering Research Center of Immunological Products, Third Military Medical University, Chongqing 400038, China

Materials

Triethanolamine (TEAH3), tetraethyl orthosilicate (TEOS), 3- bis(3-(triethoxysilyl) propyl) diselenide (BTESePD), Fluorescein Isothiocyanate (FITC) and succinic anhydride were purchased from Sigma-Aldrich Co. (St Louis, MO, USA). Other chemical reagents including sodium borohydride (NaBH4), anhydrous sodium sulfate, ammonium nitrate (NH4NO3), hydrogen peroxide (30% H2O2) and anhydrous ethanol were purchased from Beijing Chemical Reagent Co. (Beijing China). DiR and 4',6-diamidino-2-phenylindole (DAPI) were purchased from Thermo Fisher Scientific Inc. (NYSE: TMO). anti-CD3-PerCP/cy5.5, anti-CD8a-APC, anti-CD11c-FITC, anti-CD86-PE, and anti-CD80-APC were purchased from Abcam. PE-labelled H-2K^b^ OVA tetramer was purchased from MBL. Fetal bovine serum (FPS), RPMI1640 media, DMEM media and 0.25% trypsin-EDTA were purchased from Gibco Co., Ltd. TNF-α, IL-6, IL-12, IFN-γ and IFN-β ELISA kits were purchased from BioLegend Co., Ltd.

Characterization, degradation, and drug release

The morphologies of the NA and SCCM@NA samples were characterized with a JEM-2100F transmission electron microscope (TEM, JEOL, Ltd., Japan). The hydrodynamic diameter and zeta potential were analyzed using a Nano-ZS 90 Nanosensor (Malvern Instruments Ltd., Worcestershire, UK). To evaluate the stability of the SCCM@NA, the particles were stored in PBS for 7 days, after which the hydrodynamic diameter was measured via DLS every day. The expression of classical MHC-I molecules and the H-2K^b^-restricted OVA-derived peptide SIINFEKL were quantified via flow cytometry. The SASP-related factors (IL-6, IL-10, IFN-γ and TNF-α) were detected by analyzing cell suspensions with ELISA kits (BioLegend).

To assess its degradability, 2 mg of SCCM@NA was dispersed in 5 mL of PBS with or without 100 μM H_2_O_2_. These dispersions were maintained at 37°C with constant rotation. At specified time points, the samples were obtained, and their morphologies were examined via TEM. The drug release kinetics of SCCM@NA were evaluated concurrently within the same setting. The release profile of CpG within the supernatant was quantified via UV–Vis.

Collection of BMDCs

BMDCs were collected form bone marrow of C57BL/6. Bone marrow was flushed out of the bone with a 1 mL sterile syringe using warm PRIM 1640 media containing with 10% fetal bovine serum (Gibco) and 1% penicillin/streptomycin. Cells were then pelleted at 600 × g for 5 min, resuspended in BMDC growth media, consisting of the basal media further supplemented with 20 ng/mL granulocyte/macrophage-colony stimulating factor (GM-CSF; Protech). Media were half-changed every two days. On day 5, BMDCs were collected and plated 2×10^6^ cells per well into 6-well plates in BMDC growth media for following experiments.

Cytotoxicity and cellular uptake

BMDCs and RAW 264.7 cells were plated in a 96-well plate (5000 cells/well) and cultured overnight. Subsequently, various nanovaccines (from 3.12 to 200 μg mL^−1^) were added to the BMDCs and RAW 264.7 cells, which were incubated for 24 h or 48 h. Then, cell viability was determined via a CCK-8 kit according to the manufacturer's protocol.

To investigate the cellular internalization of nanovaccines by APCs, BMDCs were plated in a 24-well plate overnight and then co-incubated with FITC-labeled NA, CCM@NA, ICCM@NA or SCCM@NA at a concentration of 12.5 μg mL^−1^. After 1 h of co-incubation, the BMDCs were rinsed with PBS, fixed using 4% paraformaldehyde, stained with DAPI and subsequently observed via laser scanning confocal microscopy (CLSM). To quantitively analyze the cellular uptake, the cells were washed, trypsinized, resuspended and analyzed via flow cytometry.

Systemic toxicity evaluation

The mice were administered saline or SCCM@NA plus αPD-1 on Day 0 and 7. The body weights of the mice were measured every 2 days. The experiment was terminated on Day 60, after which the surviving mice were sacrificed. The major organs, encompassing the heart, liver, spleen, lung and kidney, were collected and subjected to HE staining, and the blood was collected for the analysis of serum biochemistry.


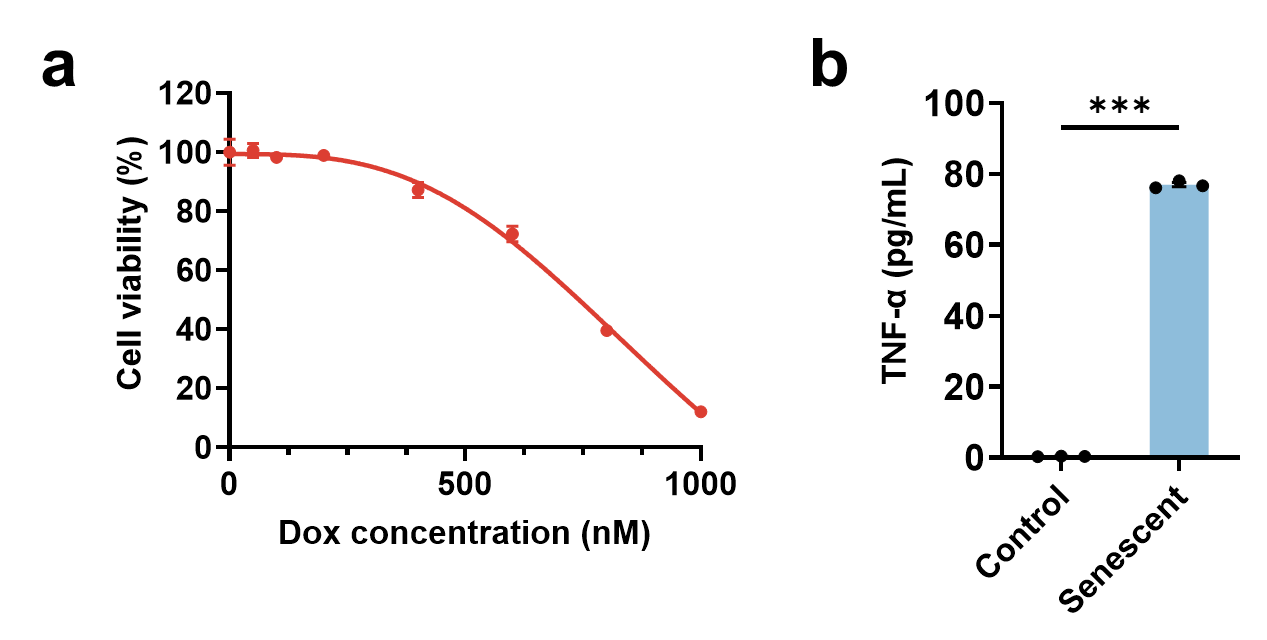


Figure S1. (a) Cytotoxicity of Dox at various concentrations on B16-OVA cancer cells for 5 days. (b) Expression levels of TNF-α in untreated or senescence-induced cell suspensions. The data represent the mean ± SD; n = 3. ****P* < 0.001.


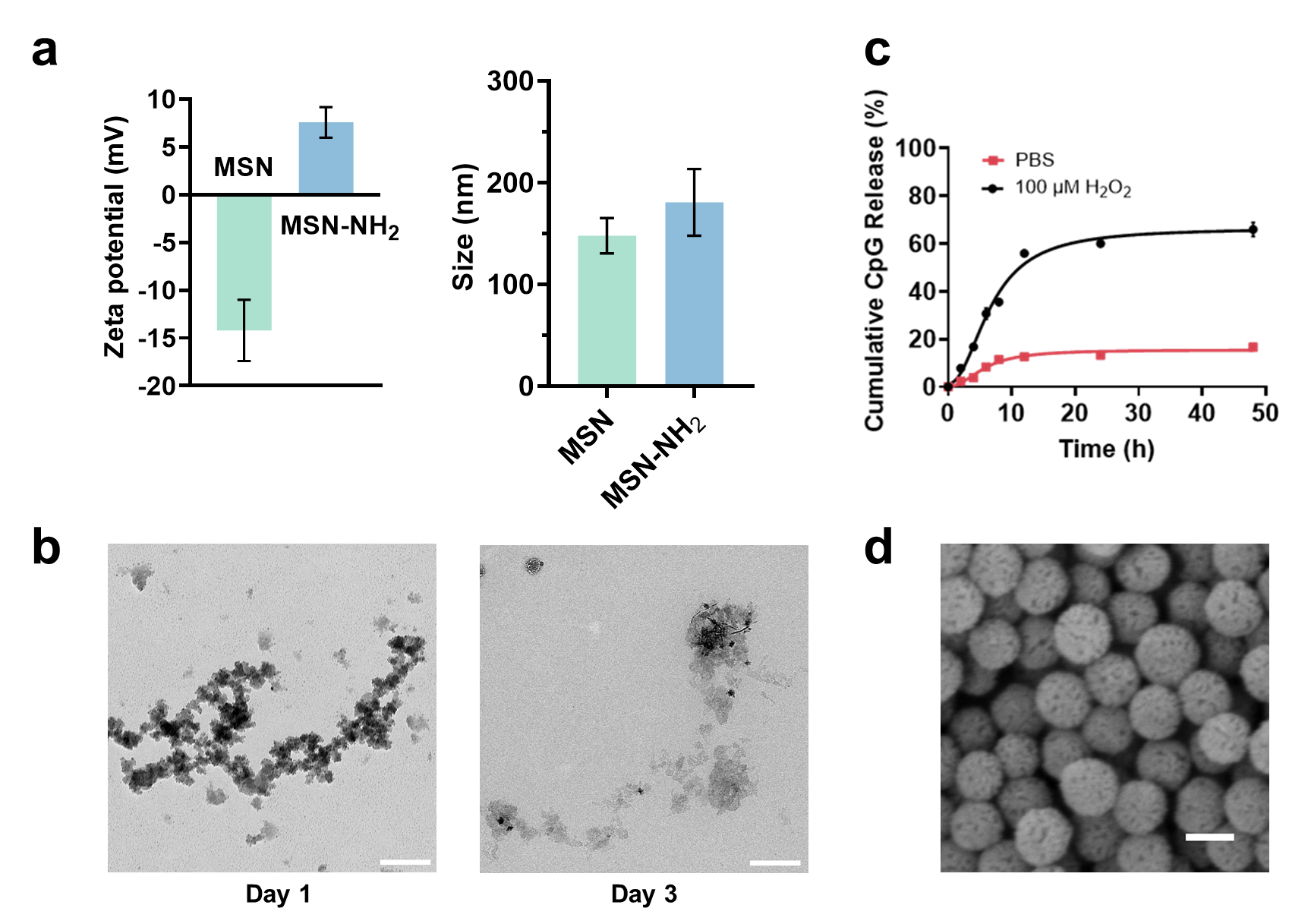


Figure S2. (a) Zeta potential and hydrodynamic size of MSN and MSN-NH_2_. (b) The morphology of SCCM@NA after immersion in 100 μM H_2_O_2_ solution for 1 and 3 days; scale bar, 500 nm. (c) Cumulative CpG release profile of SCCM@NA in PBS solution containing 0 or 100 μM H_2_O_2_. (d) The SEM image of the NA; scale bar, 200 nm. The data represent the mean ± SD; n = 3.


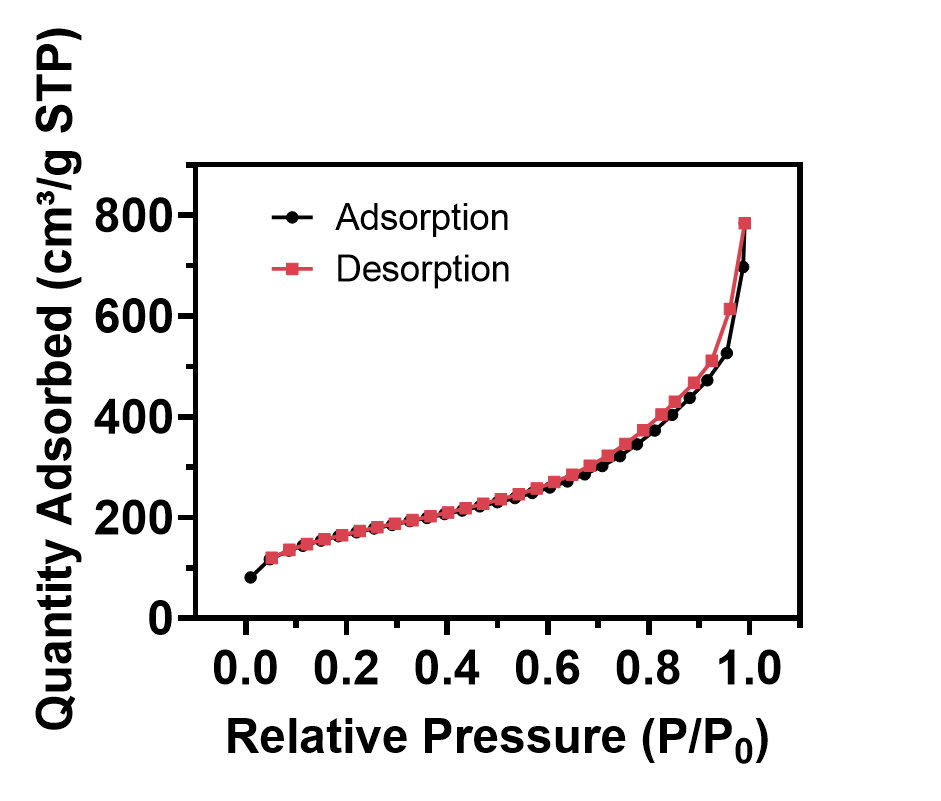


Figure S3. N_2_ adsorption−desorption isotherms of the NA.


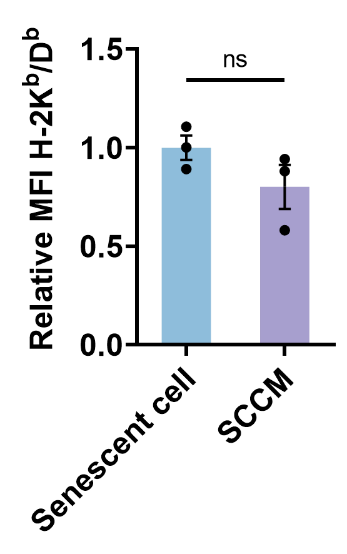


Figure S4. Flow cytometry analysis of H-2K^b^/D^b^ expression in senescence-induced B16-OVA cancer cells and SCCM vesicles. The data represent the mean ± SD; n = 3. *ns*, not significant.


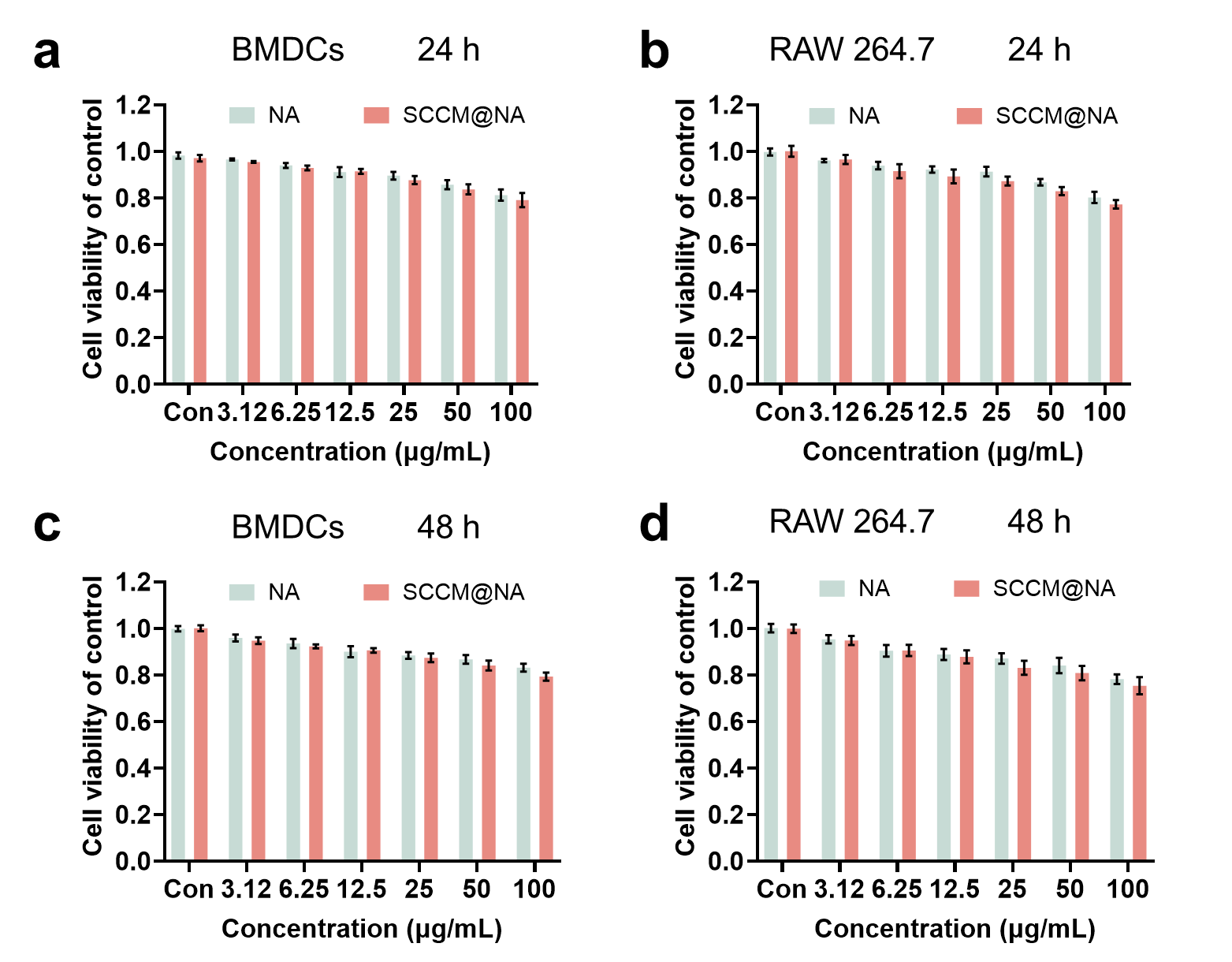


Figure S5. Cell viability of (a, c) BMDCs and (b, d) RAW 264.7 cells incubated with various concentrations of NA or SCCM@NA for 24 h (a, b) and 48 h (c, d). The data are presented as the mean ± SD; n = 4.


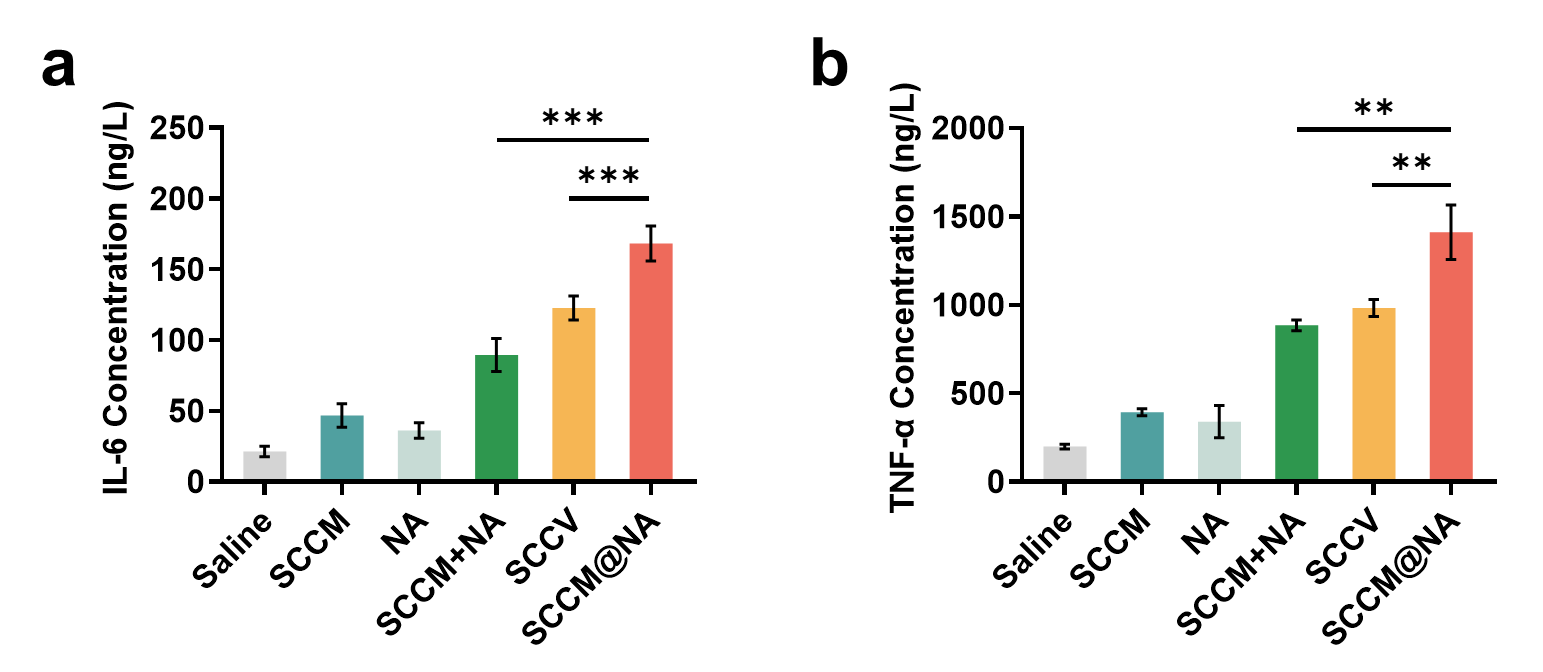


Figure S6. APCs were incubated with nanovaccines or various formulations. (a) Secretion of IL-6 in BMDC suspensions as measured by ELISA. (b) Secretion of TNF-α in macrophage (RAW 264.7) suspensions as measured by ELISA. The data represent the mean ± SD; n = 3. ***P* < 0.01, and ****P* < 0.001.


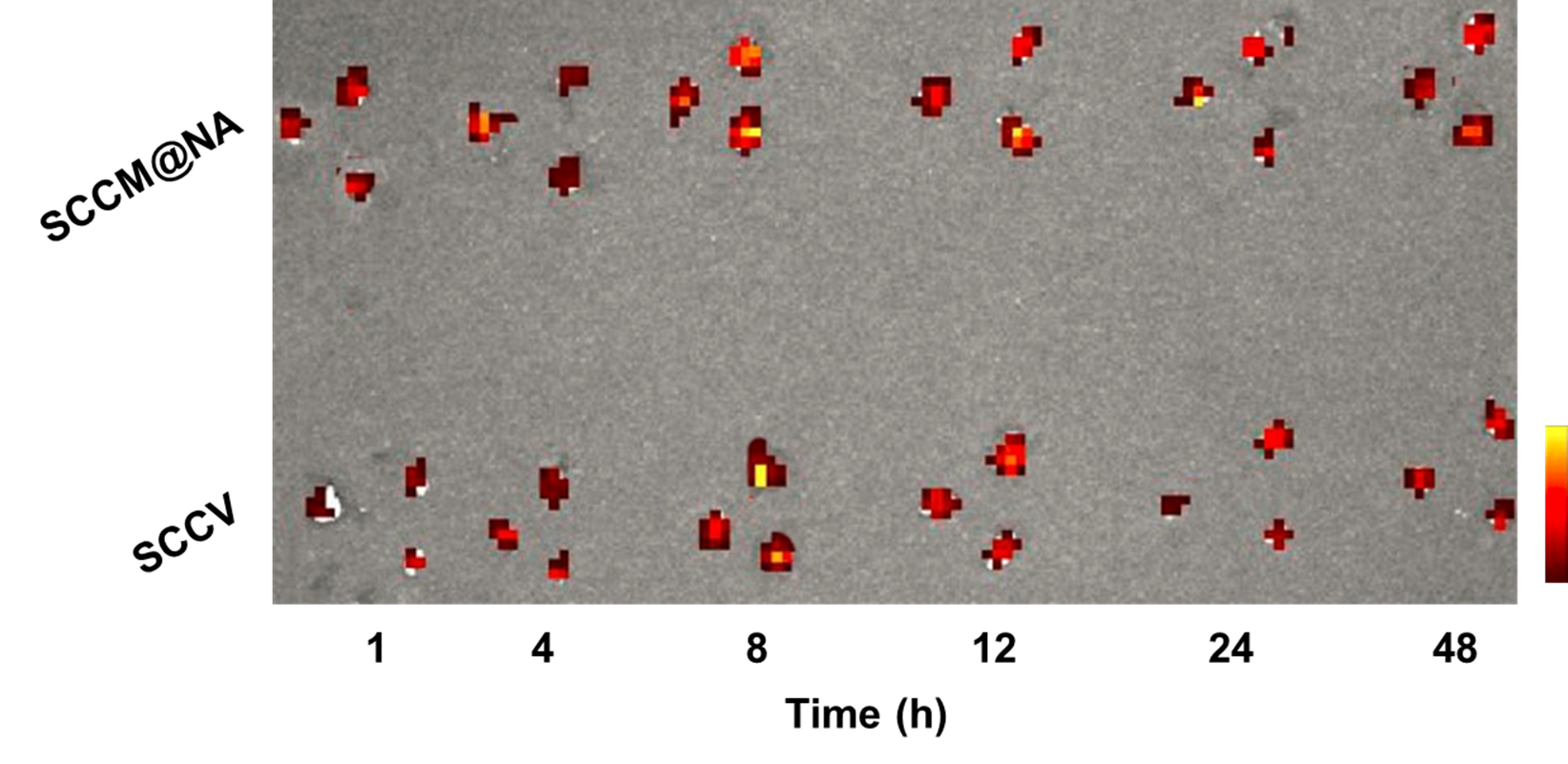


Figure S7. Fluorescence images of popliteal lymph nodes treated with DiR-labeled membrane-derived vaccines at the indicated time points.


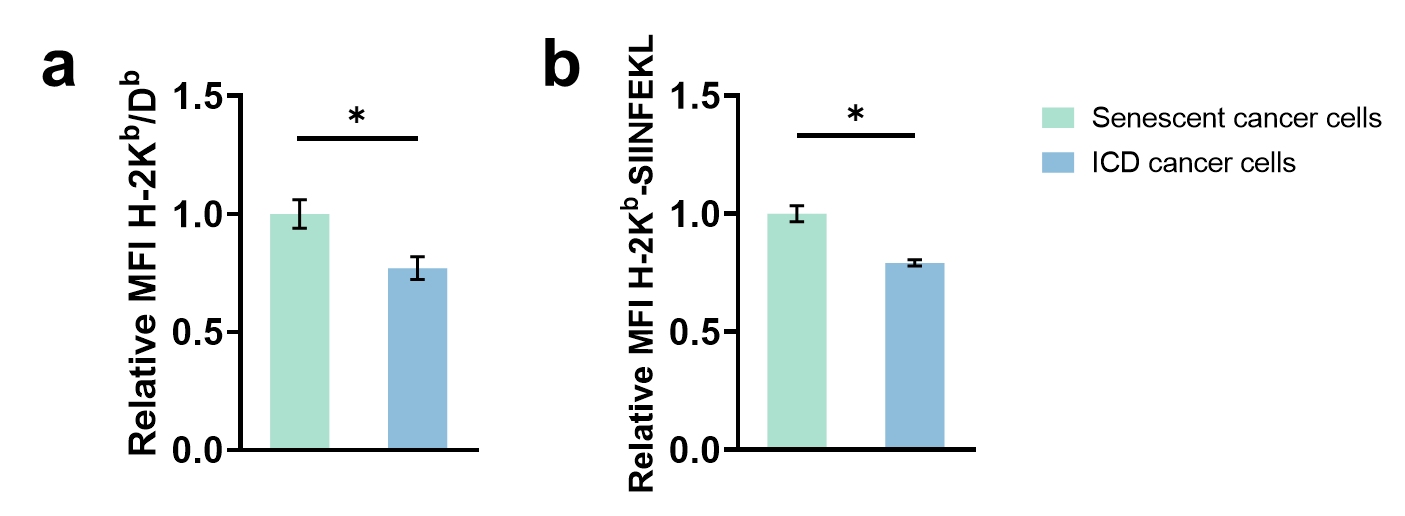


Figure S8. Flow cytometry analysis of H-2K^b^/D^b^ (a) and the presentation of the OVA-derived peptide SIINFEKL (b) in senescence-induced B16-OVA cancer cells and ICD-induced B16-OVA cancer cells. All data are mean ± SD; n = 3. **P* < 0.05.


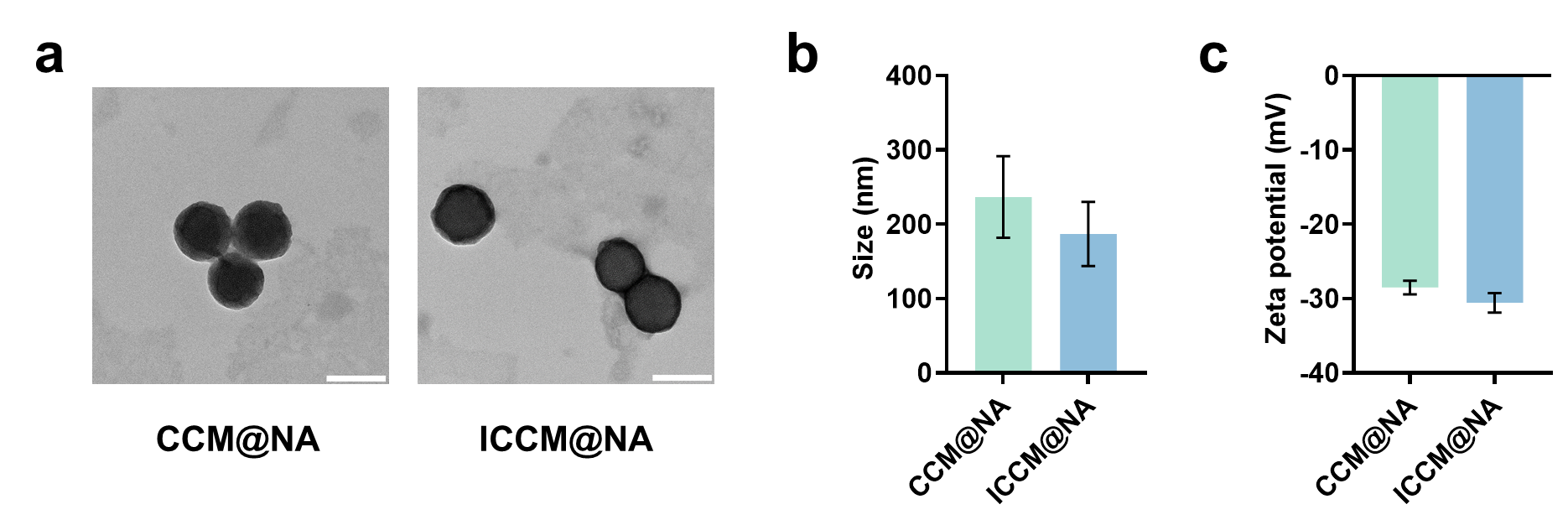


Figure S9. Characterization of the CCM@NA and ICCM@NA. (a) TEM images of CCM@NA (left) and ICCM@NA (right); scale bar, 200 nm. The hydrodynamic size (b) and zeta potential (c) of CCM@NA and ICCM@NA. The data represent the mean ± SD; n = 3.


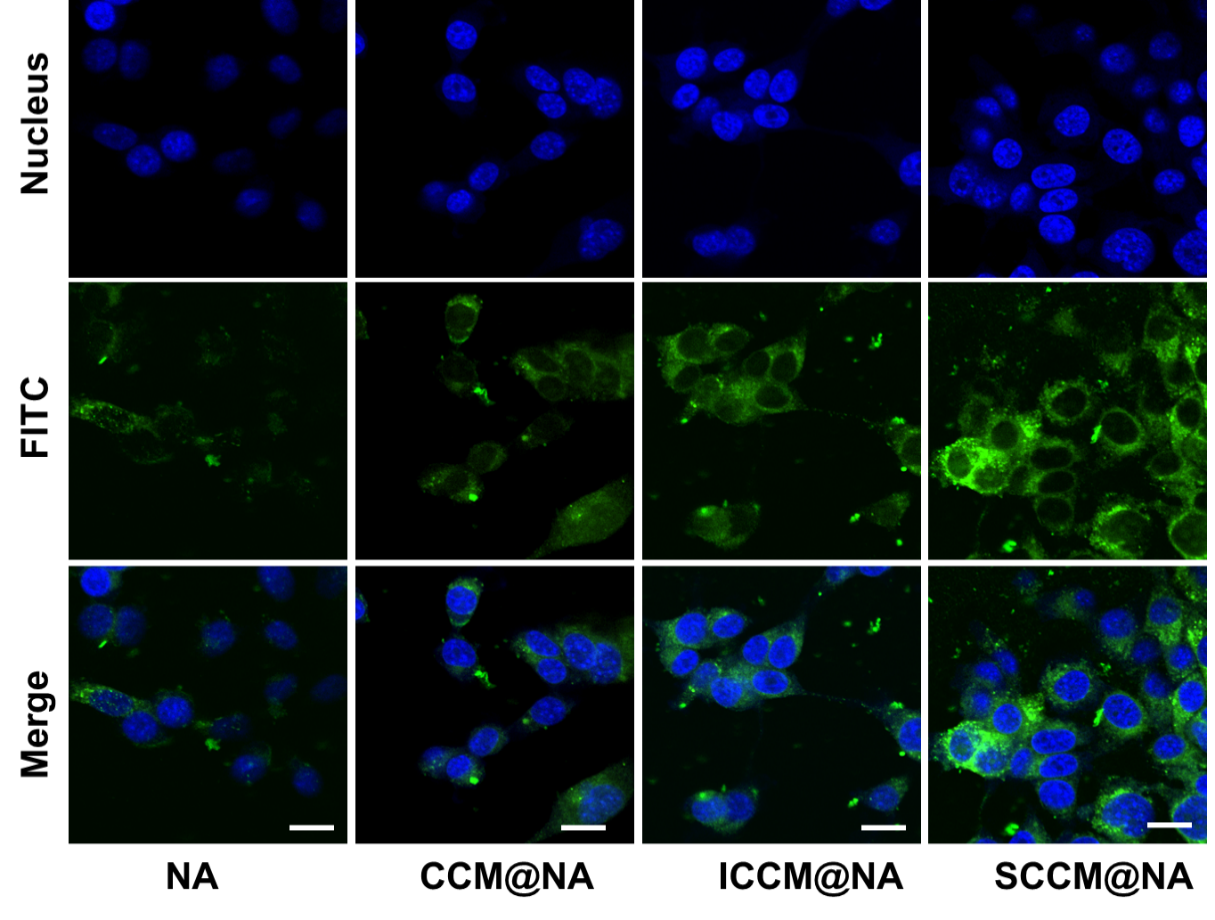


Figure S10. Cellular internalization of FITC-labeled NA, CCM@NA, ICCM@NA and SCCM@NA after 1 h of incubation with BMDCs. Scale bar, 10 μm.


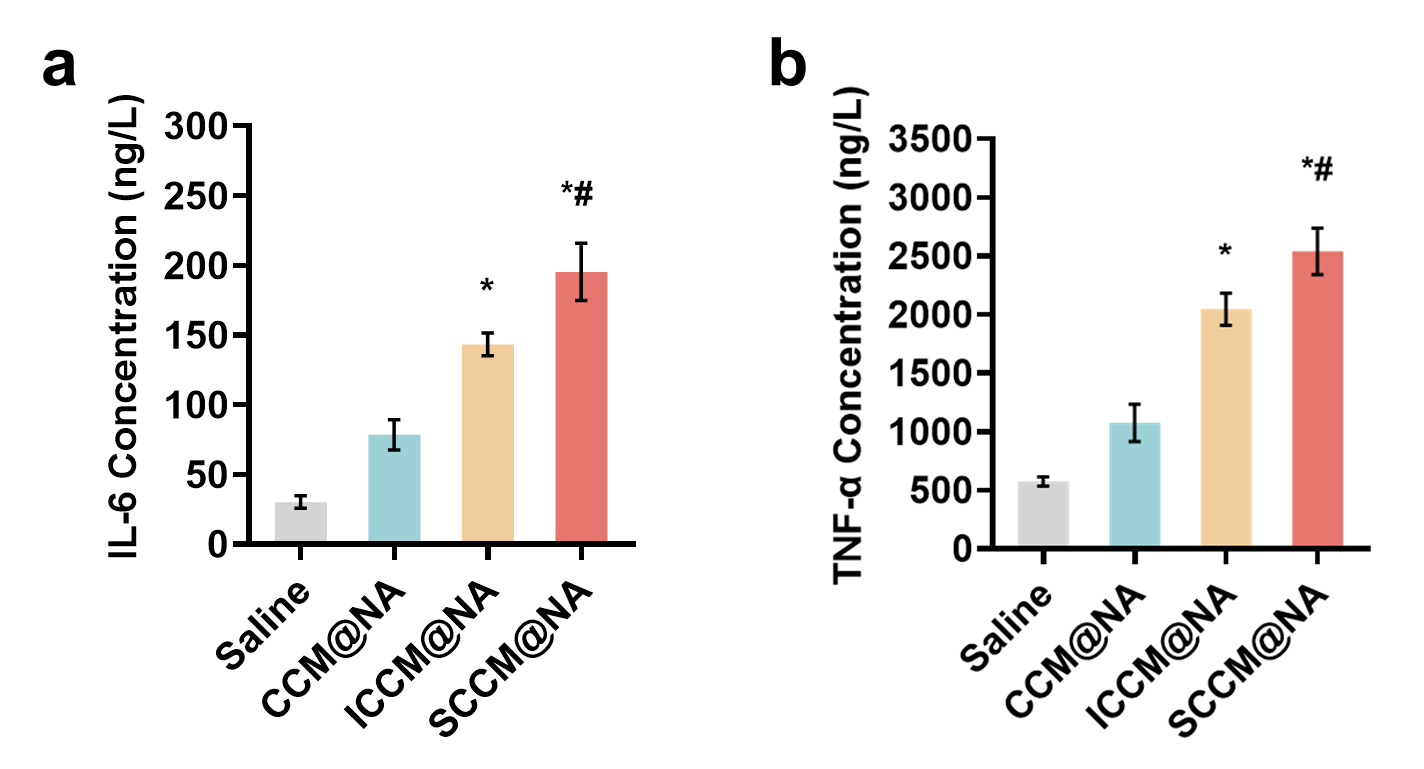


Figure S11. Secretion of IL-6 and TNF-α in BMDC suspensions as measured by ELISA. The data represent the mean ± SD; n = 3. **P* < 0.05 vs CCM@NA group, #*P* < 0.05 vs ICCM@NA group.


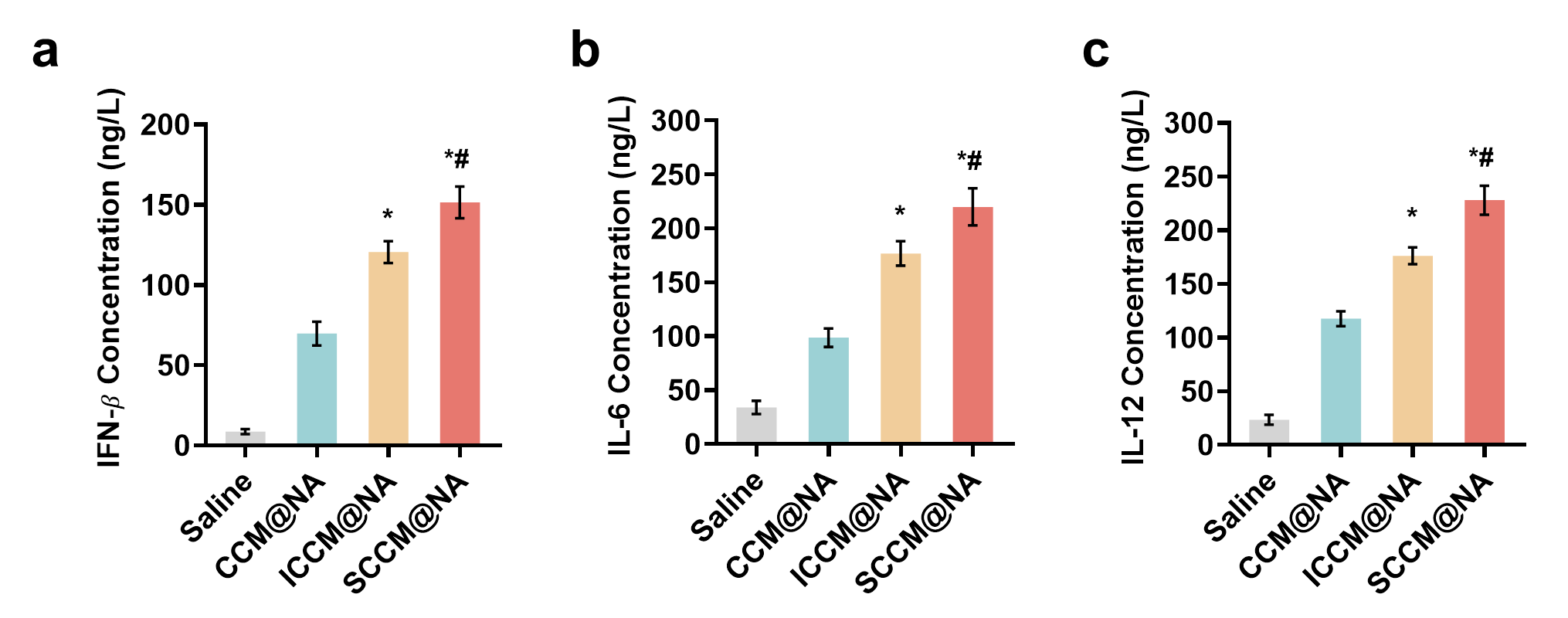


Figure S12. Secretion of IFN-β, IL-6 and IL-12 as measured by ELISA. The data represent the mean ± SD; n = 3. **P* < 0.05 vs CCM@NA group, #*P* < 0.05 vs ICCM@NA group.


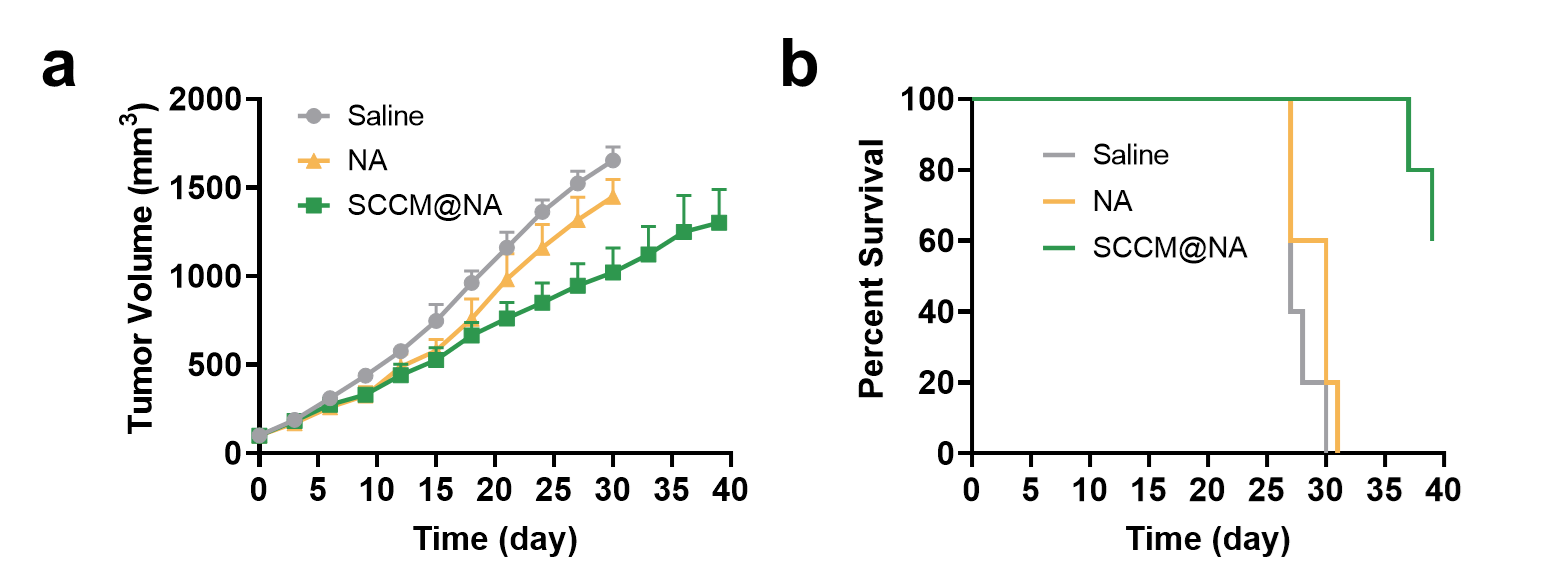


Figure S13. The therapeutic effects of NA and SCCM@NA. (a) Quantitative assay of tumors volume (n = 5). (b) Survival profiles of mice after immunization with different formulations (n = 5). All data are mean ± SD.


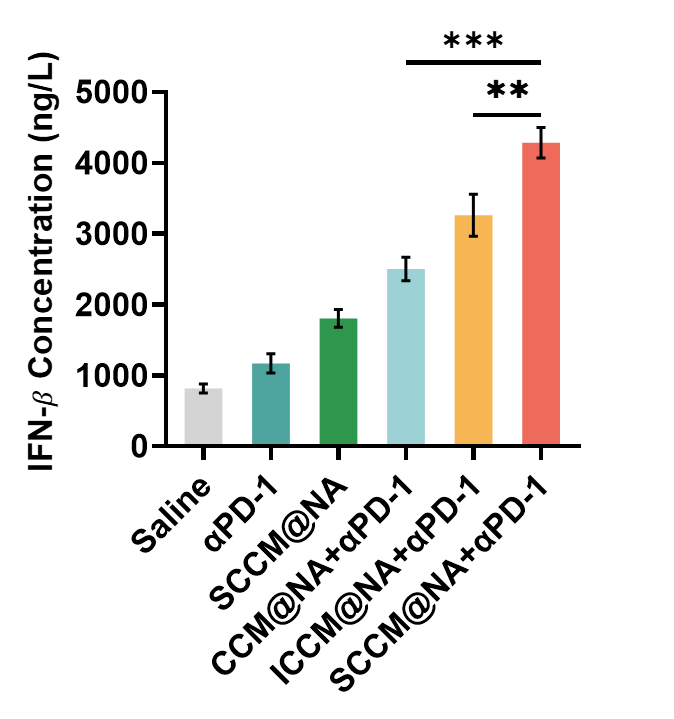


Figure S14. Secretion of IFN-β as measured by ELISA. The data represent the mean ± SD; n = 3. ***P* < 0.01, and ****P* < 0.001.


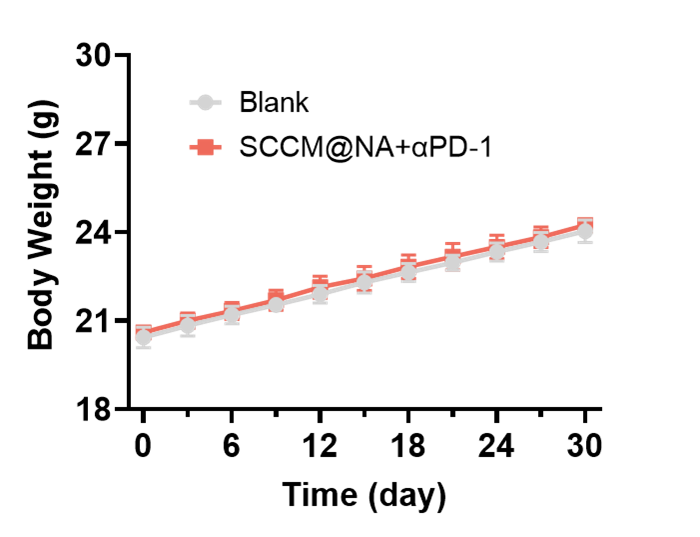


Figure S15. Body weight of mice in the SCCM@NA+αPD-1 group. The data represent the mean ± SD; n = 5.


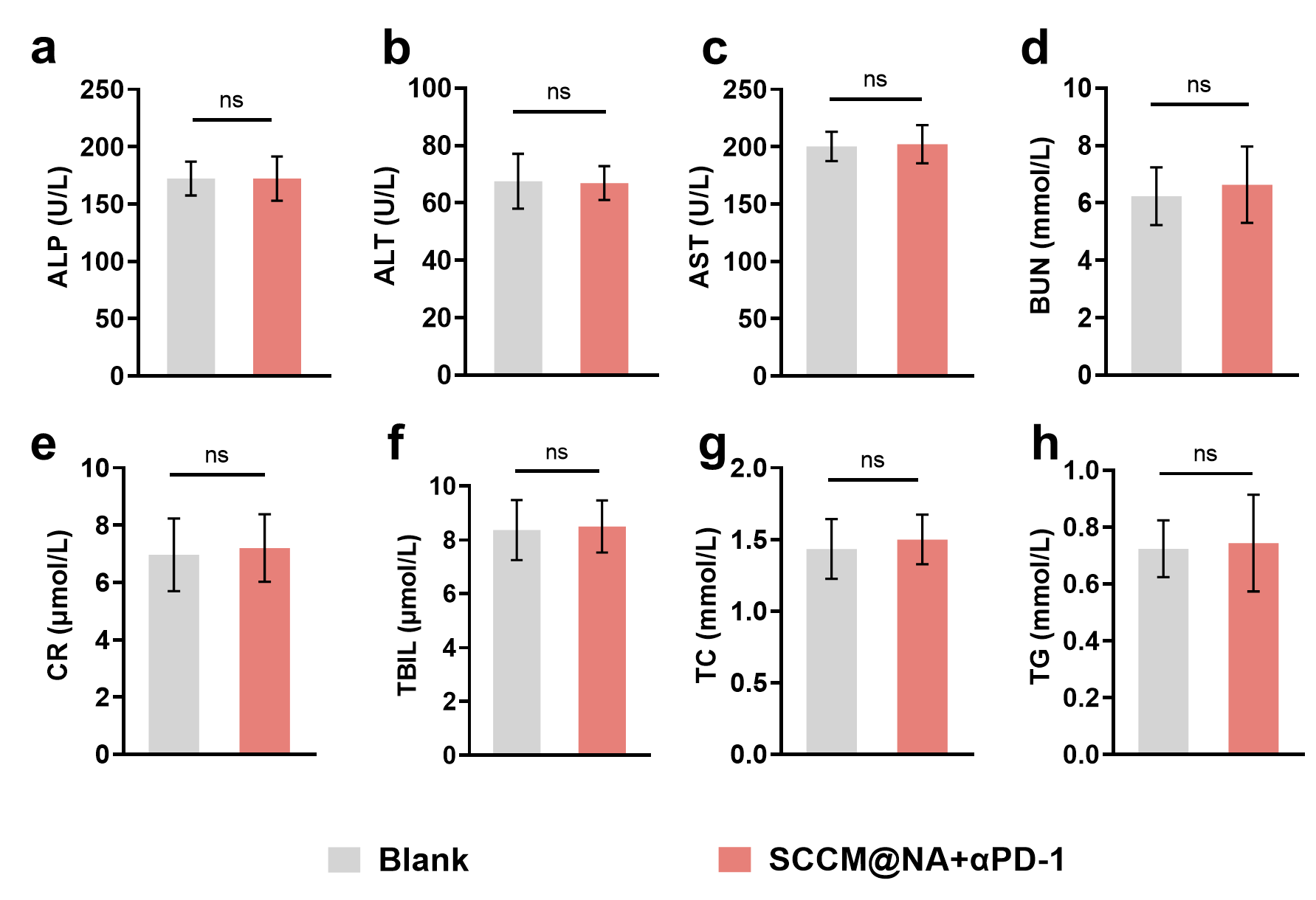


Figure S16. Serum biochemical parameters, including (a) ALP, (b) ALT, (c) AST, (d) BUN, (e) CR, (f) TBIL, (g) TC, and (h) TG of mice in the SCCM@NA+αPD-1 group on Day 60. The data are presented as the mean ± SD; n = 5. *ns*, not significant.


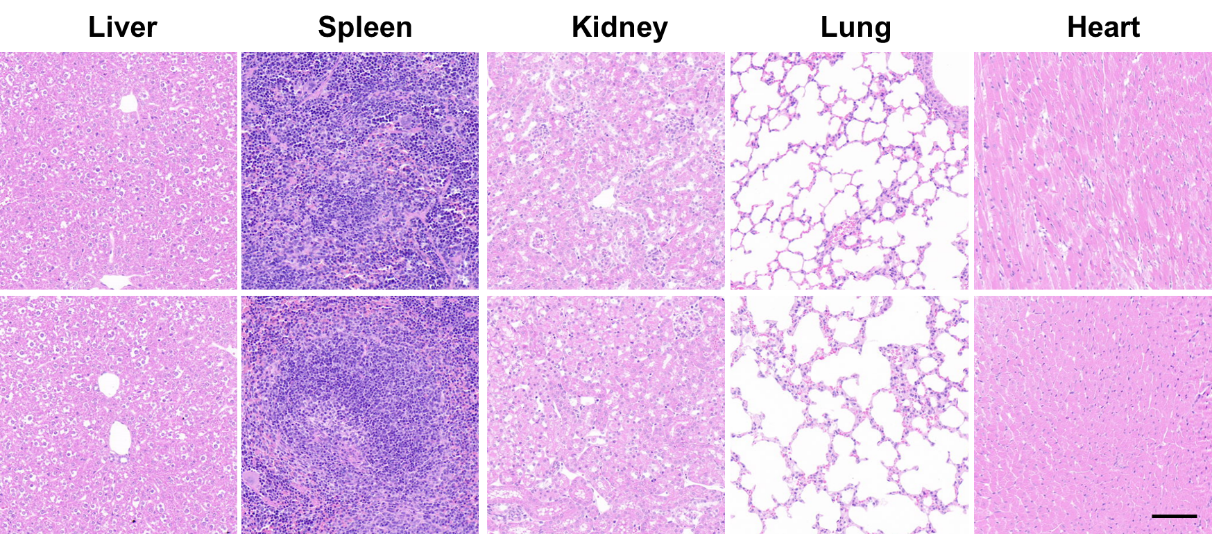


Figure S17. Representative H&E staining images of the heart, liver, spleen, lung, and kidney of the SCCM@NA+αPD-1 group on Day 60. Scale bar, 100 μm.
